# Marine bacteroidetes use a conserved enzymatic cascade to digest diatom β-mannan

**DOI:** 10.1038/s41396-022-01342-4

**Published:** 2022-11-21

**Authors:** Irena Beidler, Craig S. Robb, Silvia Vidal-Melgosa, Marie-Katherin Zühlke, Daniel Bartosik, Vipul Solanki, Stephanie Markert, Dörte Becher, Thomas Schweder, Jan-Hendrik Hehemann

**Affiliations:** 1 Pharmaceutical Biotechnology, Institute of Pharmacy, University Greifswald, 17489 Greifswald, Germany; 2 Max Planck Institute for Marine Microbiology, 28359 Bremen, Germany; 3 University of Bremen, Center for Marine Environmental Sciences, MARUM, 28359 Bremen, Germany; 4 Institute of Marine Biotechnology, 17489 Greifswald, Germany; 5 Institute of Microbiology, University Greifswald, 17489 Greifswald, Germany

**Keywords:** Water microbiology, Proteomics, Microbial ecology

## Abstract

The polysaccharide β-mannan, which is common in terrestrial plants but unknown in microalgae, was recently detected during diatom blooms. We identified a β-mannan polysaccharide utilization locus (PUL) in the genome of the marine flavobacterium *Muricauda* sp. MAR_2010_75. Proteomics showed β-mannan induced translation of 22 proteins encoded within the PUL. Biochemical and structural analyses deduced the enzymatic cascade for β-mannan utilization. A conserved GH26 β-mannanase with endo-activity depolymerized the β-mannan. Consistent with the biochemistry, X-ray crystallography showed the typical TIM-barrel fold of related enzymes found in terrestrial β-mannan degraders. Structural and biochemical analyses of a second GH26 allowed the prediction of an exo-activity on shorter manno-gluco oligosaccharides. Further analysis demonstrated exo-α-1,6-galactosidase- and endo-β-1,4-glucanase activity of the PUL-encoded GH27 and GH5_26, respectively, indicating the target substrate is a galactoglucomannan. Epitope deletion assays with mannanases as analytic tools indicate the presence of β-mannan in the diatoms *Coscinodiscus wailesii* and *Chaetoceros affinis*. Mannanases from the PUL were active on diatom β-mannan and polysaccharide extracts sampled during a microalgal bloom at the North Sea. Together these results demonstrate that marine microorganisms use a conserved enzymatic cascade to degrade β-mannans of marine and terrestrial origin and that this metabolic pathway plays a role in marine carbon cycling.

## Introduction

Polysaccharides are abundant biomolecules in the marine carbon cycle. By combining various monosaccharides, glycosidic linkage configurations, connections and functional groups, polysaccharides create molecular diversity [1]. They act as energy storage, cell wall components, extracellular defense and communication agents [2–6]. To yield energy and carbon from the diversity of existing polysaccharides, microorganisms use a proportionally complex arsenal of carbohydrate-active enzymes (CAZymes) targeting the glycosidic linkages and other chemical groups. CAZymes include the enzyme classes glycoside hydrolases (GHs) and carbohydrate esterases (CEs), which are further classified in families according to the CAZy database by their sequence, fold and substrate specificity [7]. CAZymes catalyzing degradation of a type of polysaccharide are often organized in genomic islands, named polysaccharide utilization loci (PULs). Alongside CAZymes, these clusters contain genes for specific uptake and recognition (SusC- and SusD-like) proteins as well as transcriptional regulators [8]. Together, they form highly specific uptake machineries for a multitude of substrates.

Members of the phylum *Bacteroidetes* have been studied as polysaccharide degraders of terrestrial ecosystems especially in the human gut [8, 9]. They are also prominent in aquatic habitats, especially during marine algae blooms. Microalgae bloom in near-surface, nutrient-rich regions and contribute to the global carbon cycle by fixing carbon on a scale roughly equal with terrestrial phototrophs [10, 11]. Most of this biomass is re-mineralized by heterotrophic bacteria, such as members of the *Flavobacteriia* within the *Bacteroidetes*. Metagenomics and metaproteomics of such bacterioplankton revealed a genetic potential for the decomposition of structurally diverse polysaccharides [12–14]. Although this genetic potential appears remarkably similar to human gut bacteroidetes and their well-studied role in food polysaccharide breakdown, many marine polysaccharides relevant in microalgal blooms remain to be characterized.

Recently, we discovered over 20 different polysaccharide structures in particulate organic matter (POM) and high molecular weight dissolved organic matter (HMWDOM) during spring blooms around the North Sea island of Helgoland [15], including a β-1,4-mannan. β-Mannans are a complex group of glycans found in plants as homo- and heteropolymers. While homomannan consists of a linear backbone of β-1,4-linked mannose [16], β-mannans can also exists as a backbone of β-1,4-linked mannose and glucose, known as glucomannan [17]. Both linear mannan and glucomannan can have α-1,6-galactosyl branch points and are then referred to as galactomannan and galactoglucomannan, respectively [18]. Additionally, some mannans are acetylated at O2 or O3 of the mannose monomer [19, 20].

The presence of mannan during microalgae blooms suggested that this substrate might be the potential target for a PUL type with multiple GH26 β-mannanases previously identified in marine isolates (*Salegentibacter* sp. Hel_1_6, *Leeuwenhoekiella* sp. MAR_2009_132 and *Sediminibacter* sp. Hel_1_10). These bacteria were shown to also consume terrestrial mannan, leading to the expression of PULs related to those in the human gut symbiont *B. ovatus* specific for galactomannan [21, 22]. The marine PULs, however, have additional enzymes such as a predicted GH27 α-galactosidase and a predicted glucanase (GH5), indicating adaptation to a more complex polysaccharide. Here we show that the marine *Muricauda* sp. MAR_2010_75 strain possesses a conserved PUL, which is specifically induced by β-mannan. The core enzymes of the PUL catalyze β-mannan utilization and can degrade β-mannan from HMWDOM sampled during a diatom bloom at the North Sea as well as β-mannans extracted from diatoms of the genera *Coscinodiscus* and *Chaetoceros*, both relevant to marine microalgae blooms.

## Materials and methods

### Comparative genomics

RefSeq assemblies of genomes deposited in MarRef (v1.7) and MarDB (v1.6) [23] were downloaded from NCBI web server [24] and screened for co-occurrence of *Muricauda* sp. MAR_2010_75 β-mannan PUL coding sequences using the “hmm” search function of cblaster (v1.3.14) [25] with the HMM profiles “TIGR04056” (SusC-like), “PF12741.10”, “PF12771.10”, “PF14322.9” and “PF07980.14” (all SusD-like) as well as “GH5”, “GH26”, “GH27”, “GH130” and “CE2” from the dbCAN-HMMdb-V10 database [26]. Assemblies containing putative clusters with at least one GH26 and a SusCD-pair were kept for further analysis. CAZymes were annotated using the hmmscan function of HMMer (v3.3.2) against the dbCAN-HMMdb-V10 database. Results were parsed using the hmmscan-parser.sh script provided by dbCAN and additionally confirmed using Protein-Protein BLAST (v2.11.0 + ) [27] against the CAZyDB (release 09242021) with an e-value threshold of E-20, minimum query coverage of 40% and at least 30% sequence identity [28]. PULs were extracted by screening the genetic context of SusCD-pairs for CAZyme annotations using a seven-gene frame, excluding glycosyl transferases. SusC/D genes and CAZyme repertoire of previously published MAGs from 2010 to 2012 and 2016 (available in ENA project PRJEB28156, see [29] for details) were predicted as described above, regardless of the genetic context. Results were visualized with UpSetR [30, 31] and Circos [32]. Synteny between selected reference PULs and the *Muricauda* sp. MAR_2010_75 PUL were analyzed using Protein-Protein BLAST with an e-value threshold of E-5. For phylogenetic analysis of taxa encoding β-mannan targeting PULs, *rpoB*-sequences were aligned using T-Coffee web service of EMBL-EBI [33] (v13.41.0.28bdc39) with default settings. Maximum-likelihood phylogeny was estimated by PhyML 3.0 [34] using automatic model selection by SMS [35] with Bayesian Information Criterion. The resulting tree was visualized using iTOL [36].

### Diatom isolation

An algae net sample with a cut-off of 80 µm was collected from the Helgoland Bight (54˚11.3′N, 7˚54.0′E) in 2017. From this sample, *Coscinodiscus wailesii* cells were transferred to 24 well plates (Thermo Fisher Scientific, Waltham, MA, USA) in F/2 media at room temperature (RT) with a 12:12 h light:dark cycle and transferred until no other diatoms were growing in co-culture. Once in monoculture, they were routinely cultured in 25 mL tissue culture flasks under the same conditions.

### Strain and cultivation conditions

*Muricauda* sp. MAR_2010_75 was isolated as previously reported [14, 37]. Growth experiments were performed in synthetic seawater medium (MPM) [38] with 0.2% (w/v) defined carbon sources at 21 °C and 200 rpm. Growth was determined by optical density (600 nm). As homomannan is insoluble in water, growth on this substrate was determined via protein concentration (Pierce BCA Protein Assay Kit, Thermo Fisher Scientific, Waltham, MA, USA).

### Proteomics

Cultures of *Muricauda* sp. MAR_2010_75 grown to late exponential phase with glucomannan, galactomannan, homomannan, mannose and citrus pectin (control) as sole carbon source were used for proteome analyses. All substrates were purchased from Megazyme. Details of protein extraction and subproteome enrichment can be found in the Supplementary Information.

Peptides were separated using reversed phase C18 column chromatography on a nano ACQUITY-UPLC (Waters Corporation, Milford, MA, USA) online-coupled to an LTQ-Orbitrap Classic mass spectrometer (Thermo Fisher Scientific Inc., Waltham, MA, USA) [39]. Spectra were searched against a target-decoy protein sequence database including sequences and reverse sequences of *Muricauda* sp. MAR_2010_75 and of common laboratory contaminants using MaxQuant [40], applying a protein FDR as well as peptide level FDR of 0.01 (1%). Only proteins that could be detected in at least two of three replicates were considered identified. Automatically calculated iBAQ values (intensity-based absolute quantification) were used to manually calculate % riBAQ values (relative iBAQ; giving the relative protein abundance of all proteins in the same sample) for semiquantitative comparisons between samples from different conditions. Tests for differential expression were performed using Perseus v. 1.6.2.3 [41] with Welch’s two-sided t-test (permutation-based FDR 0.05). Subcellular protein location was deduced using subproteome data combined with PSORTb 3.0 [42] and CELLO [43] analysis. Data and Results are available through the ProteomeXchange Consortium (http://proteomecentral.proteomexchange.org) via the PRIDE partner repository [44] with the identifier PXD033586.

### Diatom and HMWDOM polysaccharide extraction and microarray analysis

Diatom species (Table S1) were grown in lab cultures as previously described [15], with two 1 L non-axenic monospecific cultures per strain. Diatom cultures were harvested 10 days after inoculation by centrifugation at 6800 x *g* for 20 min at 15 °C. Cell pellets were freeze dried, homogenized using a pestle, and alcohol precipitation was performed to obtain the alcohol-insoluble residue (AIR) enriched in polysaccharides. Polysaccharides from the diatom dry biomass (AIR) were sequentially extracted with MilliQ water, 50 mM EDTA and 4 M NaOH with 0.1% w/v NaBH_4_ as previously described [15].

Each extract was printed in duplicates onto nitrocellulose membrane (pore size of 0.45 µm, Whatman, UK) using a microarrayer (Sprint, Arrayjet, UK) obtaining several identical microarrays populated with all the diatom extracts. Two polysaccharide standards, fucoidan from *Laminaria* sp. (Glycomix) and glucomannan from *Amorphophallus konjac* (Megazyme), were dissolved in MilliQ (0.5 mg/mL) and included in the print as controls.

For the HMWDOM samples, sampling was performed in 2016 during a spring microalgae bloom period (over 2.5 months) at the North Sea (54°11.3′N, 7°54.0′E) near the island of Helgoland, Germany. 100 L of seawater (1 m depth) were filtered through 0.2 μm pore size polycarbonate filters and the filtrate concentrated by tangential flow filtration using 3 filter cassettes with a cut-off of 1 kDa. Specifics on HMWDOM field sampling, processing of the samples, polysaccharide extraction and microarray printing of the HMWDOM extracts is described in [15, 21]. Details of microarray enzymatic treatment for diatom and HMWDOM arrays as well as microarray analysis can be found in Supplementary Information.

### Cloning, protein expression and purification by chromatography

The genes for GH26A (WP_036379595.1), GH26B (WP_197062540.1), GH26C (WP_036379585.1), GH5_26 (WP_197062539.1) and GH27 (WP_036379578.1) were amplified from genomic DNA without their signal peptides by PCR using gene-specific primers (Table S2) (Biomers, Ulm, Germany) and cloned using Gibson assembly in *Escherichia coli* DH5α (New England Biolabs, Ipswich, MA, USA). Clones were in house sequenced by Sanger sequencing using Bigdye (Thermofisher, Waltham, MA, USA). For protein production, plasmid DNA was transformed into *E. coli* BL21 (DE3) (New England Biolabs, Ipswich, MA, USA) and the proteins were produced in 1 L batches of autoinduction media (ZYP5052) incubated at 20 °C for 4 days [45]. Cells were harvested by centrifugation and stored at −20 °C until processing. Cell lysis was conducted by chemical lysis. Frozen cell pellets were resuspended in 20 mL sucrose solution (25% sucrose, 50 mM TRIS (pH 8.0)). Lysozyme was added at a concentration of 1 mg/mL and the sample was incubated 10 min at room temperature with spinning. 40 mL deoxycholate solution (1% deoxycholate, 1% Triton X-10, 100 mM NaCl) was added followed by MgCl_2_ to a final concentration of 1 mM and DNase to a concentration of 1 mg/mL. The resulting lysate was centrifuged at 16,000 x *g* for 45 min at 4 °C. For purification, clarified lysate was applied to a 5 mL prepacked IMAC column (GE Healthcare Life Sciences, Marlborough, MA, USA) previously equilibrated in Buffer A (20 mM TRIS (pH 8) and 500 mM NaCl) using an ÄKTA start FPLC (fast protein liquid chromatography) system (Cytiva, Marlborough, MA, USA). The column was washed extensively with Buffer A and His-tagged protein was eluted using a gradient of imidazole to 500 mM in Buffer B. Purified protein was concentrated using a stirred cell ultrafiltration device with a 10 kDa membrane. For crystallization, the concentrated protein was polished using size exclusion chromatography [HiPrep Sephacryl S200 HR column (Cytiva, Marlborough, MA, USA)] in 20 mM TRIS (pH 8) with 200 mM NaCl. Protein was concentrated to 20 mg/mL prior to further experiments.

### Crystallization, X-ray diffraction data collection, structure solution and refinement

Crystals of GH26C were obtained by hanging drop vapor diffusion with protein mixed 1:1 with a solution of 0.2 M Li_2_SO_4_, 0.1 M Bis-Tris:HCl (pH 5.5), 24% w/v PEG3350 and incubating at 16 °C. Crystals of GH26A were obtained in an identical manner but the well solution was composed of 0.15 M MgCl_2_, 0.1 M Tris:HCl (pH 8.5), 21% w/v PEG4000, 20% v/v glycerol. Crystals were cryo-protected in mother liquor supplemented up to 30% glycerol prior to freezing by being submersed in liquid nitrogen. Diffraction data were collected at DESY P11 (Hamburg, Germany), processed using XDS, and Aimless in CCP4 [46, 47]. Molecular replacement was carried out using Phaser using the coordinates of *Cellvibrio japonicus* CjGH26C (pdb id: 2VX6) [48] for GH26C and *Podospora anserina* GH26-CBM35 (pdb: 3ZM8) for GH26A [49]. Model building was carried out automatically in Buccaneer, manually in Coot and refined using Phenix.refine and REFMAC [50–53]. Data were validated and deposited at the Protein Data Bank (PDB). Figures were made using PyMOL v.2.3.2 (Schrödinger, New York, NY, USA) [54].

### Enzyme characterization

Activity profiles of GH26A, GH26C and GH5_26 were generated using high-performance anion-exchange chromatography with pulsed amperometric detection (HPAEC-PAD). Oligosaccharide and polysaccharide standards for enzymology were acquired from Megazyme. 50 µM purified enzyme was incubated at RT in 50 mM phosphate buffer containing 50 mM NaCl and 1% w/v substrate for 2 h. Samples were inactivated at 90 °C for 10 min and centrifuged at 13,000 rpm for 10 min to remove debris. Supernatant was diluted 1:1,000 in HPLC-grade H_2_O and products were detected using HPAEC-PAD (Dionex ICS-5000+, ThermoFischer Scientific Inc., Waltham, MA, USA). Controls for each substrate containing no enzyme were treated in similar fashion.

## Results

### β-mannan PULs are a specialized adaptation in marine habitats

To examine marine β-mannan degradation we used *Muricauda* sp. MAR_2010_75 as a model organism. This bacterium was isolated from seawater samples taken at the island of Sylt in the German Bight. The 4.4 Mbp genome (GCF_000745185.1) contains a β-mannan PUL related to those in two distant marine relatives that were recently examined using proteomics without further biochemical characterization [22]. While significant PUL rearrangements compared to *Muricauda* sp. are visible in these and other recently isolated bloom-associated strains (*Salegentibacter* sp. Hel_1_6, *Leeuwenhoekiella* sp. MAR_2009_132, *Sediminibacter* sp. Hel_1_10, *Flavimarina* sp. Hel_I_48) [14], they all contain at least two GH26 β-mannanases and a GH130 β-1,4-mannosylglucose phosphorylase (Fig. S1). This presence, together with a putative GH5 as well as a conserved GH27 α-galactosidase and an epimerase suggests the substrate to be a mannose-rich polysaccharide also containing glucose and galactose.

**Fig. 1:**
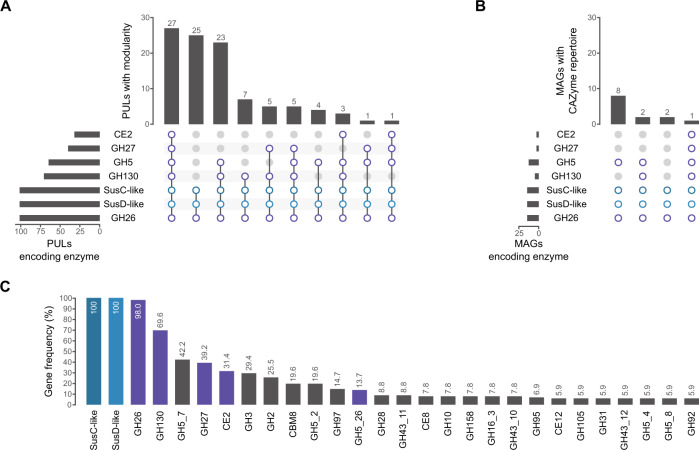
Genes for β-mannan degradation are present in marine habitats. Clusters/MAGs had to encode for at least a SusC/D pair and a GH26. CAZyme families shared with the *Muricauda* sp. PUL are colored purple, SusC/D pairs blue. **A** Modularity of different β-mannan PULs found in assemblies from marine databases. **B** MAGs from Helgoland spring bloom metagenomes containing similar CAZyme sets as the *Muricauda* sp. PUL. **C** CAZyme families contained in at least 5% of all screened β-mannan degrading clusters, highlighting their variability.

In order to analyze the relevance of β-mannan-containing substrates in marine habitats, we screened the genomes contained in publicly available marine databases (MarRef, MarDb) for clusters containing at least a SusC/D pair and a GH26. This yielded 101 clusters from 82 assemblies, 27 of which feature the same modularity as the *Muricauda* sp. PUL (Fig. 1A, Fig. S2). It also revealed a series of additional CAZymes often associated with GH26-containing clusters, including GH10, GH95 and GH97, which indicate xylose, galactose and fucose-containing substrates, respectively (Fig. 1C). The high level of co-occurrence with CAZymes not encoded within the *Muricauda* sp. PUL shows both the specificity of our PUL as well as the high variability β-mannan-containing substrates that marine habitats are likely to display.

To account for relevance during bloom situations, we additionally screened Helgoland spring bloom-associated metagenome-assembled genomes (MAGs) from 2010, 2011, 2012 & 2016 [13, 28, 29], yielding 13 MAGs that encoded for at least a SusC/D pair and a GH26. Only 1 MAG encoded for the entire repertoire of the *Muricauda* sp. PUL (Fig. 1B), where it was also ordered in a cluster. While not among the most common substrates, our results suggest β-mannan-containing polysaccharides to be relevant during microalgal blooms where they are targeted by highly specialized bacteria such as *Muricauda* sp.

### PUL is specifically upregulated by β-mannans

**Fig. 2:**
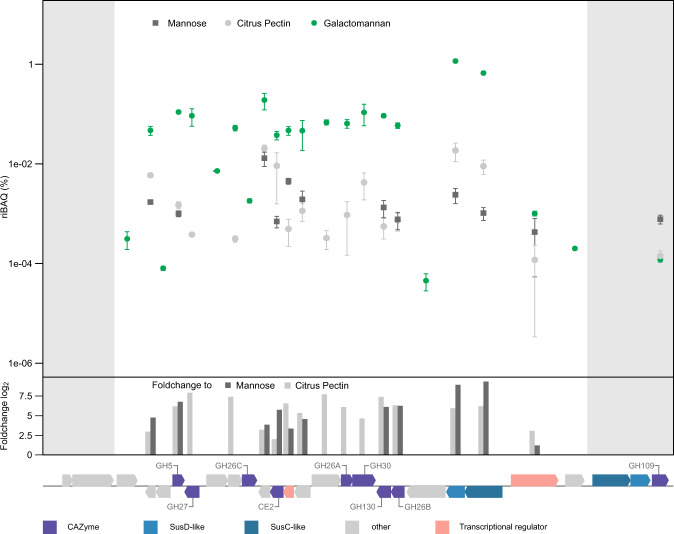
*Muricauda* sp. MAR_2010_75 PUL is specifically induced by galactomannan as sole carbon source. Abundance of PUL-encoded proteins (mean %riBAQ values and standard deviations, *n* = 3) using galactomannan, citrus pectin and mannose as substrates. Shown are the proteins FG28_RS02265-RS02395 of the β-mannan PUL. Positive foldchanges from galactomannan to other conditions are given as bars. Genes upstream and downstream of the PUL are shown for comparison (highlighted in gray).

*Muricauda* sp. MAR_2010_75 uses mannose-rich substrates as sole carbon source. This ability includes the metabolism of homo-, gluco- or galactomannan, which contain glucose in the backbone and galactose sidechains, respectively (Fig. S3). Whole cell proteomics offers experimental evidence for the genomic boundaries of the PUL and thus further indicates the conserved cluster’s function for mannan degradation. Abundance of mannan-targeting PUL-encoded proteins was increased with the three tested mannans compared to citrus pectin and the monosaccharide mannose. Except for the HTCS regulator (FG28_RS02375), the 22 proteins of our predicted β-mannan PUL (FG28_RS02275-FG28_RS02380) were significantly upregulated during growth on galactomannan with some being among the most abundant expressed proteins in the whole proteome (Fig. 2, Fig. S4 and Table S3). They represent proteins directed at mannan digestion, including necessary CAZymes as well as a SusC/D-like pair and two transcriptional regulators. Some proteins, like GH26A (FG28_RS02340), were only detected during growth on mannan. Proteins encoded by genes upstream or downstream of the PUL were either not detected in the proteome or in lower abundance compared to pectin and mannose controls (Fig. 2). This includes a SusC/D-like pair as well as a predicted α-*N*-acetylgalactosaminidase of family GH109 located directly downstream of the PUL. Thus, these proteins encoded outside of the PUL likely do not participate in mannan degradation. The proteome data demonstrate that the PUL with its 22 genes is a regulatory unit, which specifically responds to and is activated by the presence of β-mannan. As the strain grew faster and to a higher optical density on monosaccharides such as mannose (Fig. S3), it can be assumed that a bottleneck exists within the degradation cascade. Our results, which show that β-mannan degradation is performed by highly specialized bacteria (Fig. 1), make it likely that the ability to target such a complex substrate is of itself an advantage.

### PUL encodes active mannanases as well as a glucanase and galactosidase

**Fig. 3:**
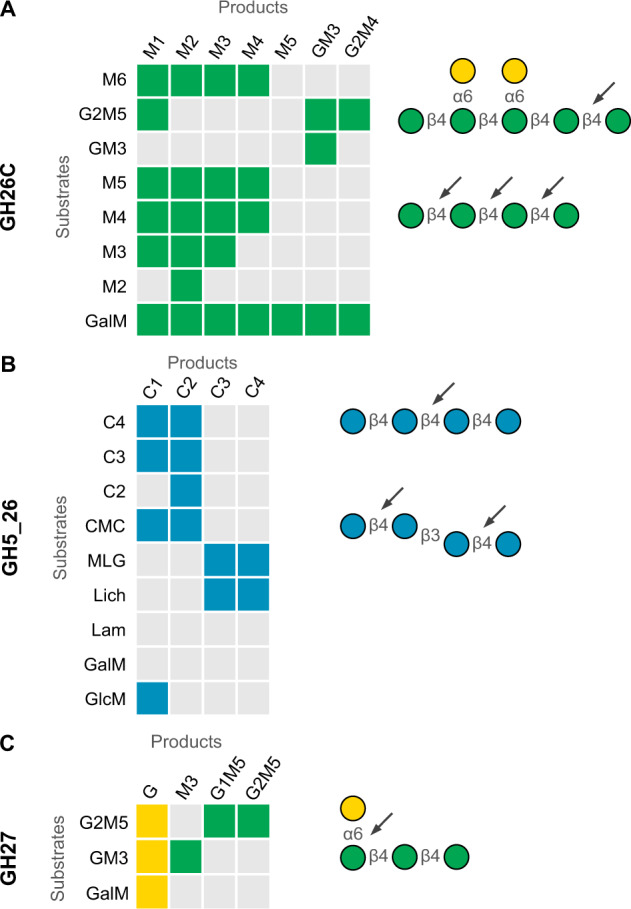
β-mannan PUL-encoded CAZymes hydrolyze different poly- and oligosaccharides. Shown are the main products formed by incubation of specific substrates with recombinantly produced **A** GH26C, **B** GH5_26 and **C** GH27 detected using HPAEC-PAD. GalM digestion with GH26C generated products of up to dp11. Oligosaccharides longer than M6 were omitted from this figure for clarity. For the full product spectrum see Fig. S5. Selected main products of each enzyme are shown in color and shape according to the Symbol Nomenclature for Glycans (SNFG) [65] and are depicted on the right. Arrows point to cleavage sites specific for each enzyme.

Consecutive stepwise β-mannan degradation begins with extracellular degradation by endo-active mannanases from GH families 26 and 5 [21]. Similarly, subproteomics combined with Cello and PSORTb analysis (Table S4) predicts that *Muricauda* sp. MAR_2010_75 displays GH26_C and GH5_26 on the extracellular surface. We recombinantly produced the extracellular endo-mannanase (GH26C) and found that it is active on β-1,4-homomannan, galactomannan and glucomannan as well as on different defined manno-oligosaccharides. Galactomannan degradation with GH26C yields a wide range of products due to the galactose branches that interfere with complete digestion (Fig. 3A). Using HPAEC-PAD we obtained peaks up to a degree of polymerization (dp) of 11. From defined manno-oligosaccharides mannose and oligosaccharides up to dp4 were released. Of the available galactosylated oligosaccharides—G2M5 and GM3—only the former is hydrolyzed, releasing mannose and a single peak for the putative product G2M4 while the latter is not affected by GH26C treatment (Fig. 3A, Fig. S5A).

As evidenced by its activity on carboxymethyl cellulose (CMC) and barley β-glucan (β-1,3/β-1,4) but not laminarin (β-1,3) or galactomannan, the predicted cellulase GH5_26 is a β-1,4-glucanase. This is in accordance with activities described for members of the GH5_26 family [55]. The enzyme hydrolyzed glucomannan, releasing glucose, consistent with activity on β-1,4-mannogluco-saccharides. On CMC and short cello-oligosaccharides dp3 and dp4, the enzyme produced a mixture of glucose and cellobiose but showed no activity on cellobiose alone. Barley glucan digestion with GH5 resulted in 4 peaks representing oligosaccharides with dp3 and dp4 containing both β-1,3- and β-1,4 linkages (Fig. 3B, Fig. S5B).

Finally, GH27 showed α-1,6-galactosidase activity as it was active on GM3 and G2M5 as well as on galactomannan, releasing galactose branches from the mannan backbone (Fig. 3C, Fig. S5C). Overall, this biochemical data shows that, although marine and terrestrial β-mannan PULs share enzymatic activities, their differences indicate the marine mannan to be more complex.

### GH26C and GH26A structures are conserved between distant protein family members

**Fig. 4:**
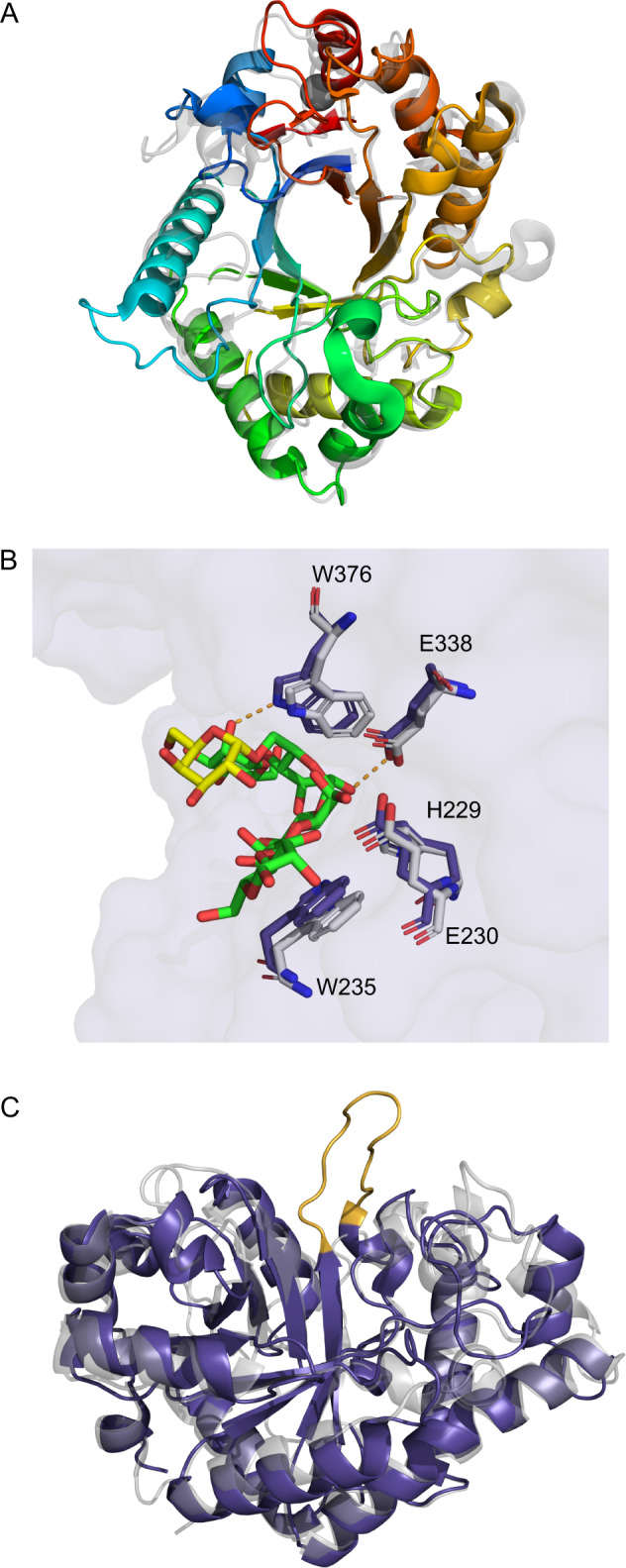
Structural analyses of *Muricauda* sp. MAR_2010_75 GH26A and GH26C allow insights into substrate binding. **A** Structure of GH26C ramped from N- to C-terminus from blue to red and overlaid with CjGH26C (2VX6, gray). **B** Active site residues of GH26C (purple) looking into the active site cleft from the side. Enzyme surface is shown in light purple. Residues are superimposed with those observed in CjGH26C (2VX6, gray) and their pentasaccharide substrate. Residues are numbered as observed in the GH26C structure. Putative hydrogen bonds stabilizing the enzyme-substrate complex are shown in orange. C-atoms belonging to mannose moieties of the bound oligosaccharide are colored green, those of galactose yellow. **C** Overview of GH26A (purple) superimposed with *P. anserina* GH26 (3ZM8, gray) looking into the active site cleft. The additional loop found in GH26A is colored orange.

Characterized glycoside hydrolases from family 26 include both mannanases and glucanases. GH26C shares only 30% sequence identity with its nearest structurally characterized homolog CjGH26C [48], so in order to determine the effects of the sequence dissimilarities, we solved the structure of GH26C to 1.50 Å from residues 47 to 420 for two chains in the asymmetric unit (Table S5). The overall structure is composed of a monomeric catalytic domain in the form of the familiar (α/β)_8_ (TIM-barrel) fold common to Clan D glycoside hydrolases (Fig. 4A) [56]. The scaffold is modified with elongated loops at the C-termini of the β-strands at the core of the structure to create a pair of lobes that form the walls of the active site groove. Modeling of the pentasaccharide G1M4 ligand from CjGH26C yielded a picture of an open active site. If anything, the groove is even larger than in CjGH26C as the BA 2 loop is orientated away from the active site groove (Fig. 4A). The key catalytic residues are conserved providing structural support for the observed biochemical activity (Fig. 4B). Overall, the marine mannanase shares a high degree of structural identity with its distant homologs from terrestrial ecosystems.

GH26A from *Muricauda* sp. MAR_2010_75 is most likely located in the periplasm (Table S4). This enzyme is 34% identical to the nearest structurally characterized GH26 (*Podospora anserina* GH26) [49] and shares the conserved catalytic residues. Nevertheless, this enzyme shows no similar activity on polysaccharides or oligosaccharide substrates. The X-ray crystal structure revealed a loop blocking the +2 subsite of the active site (Fig. 4C). These data suggest that GH26A may be a reducing end-specific exo-glycoside hydrolase. However, this does not explain why the recombinant protein is not active on the tested substrates. The structure may imply a more limited substrate specificity for which we have not yet found the correct oligosaccharide substrate such as shorter manno-gluco oligosaccharides.

A structural comparison of the two new GH26 to the enzymes available in the protein data bank reveals that they are more similar to characterized members than to each other. The closest structural homolog of GH26C is CjGH26C (2VX6) [48] with root mean square deviation (RMSD) of 1.81 Angstroms (Å) over 342 residues aligned. The closest structural homolog of GH26A is a mannanase from *Podospora anserina* (3ZM8) [49] with a C-alpha root mean square deviation of 2.41 Å over 290 residues aligned. On the other hand, M_GH26C and M_GH26A share an RMSD of only 3.12 Å over 301 residues and a sequence identity of 27%.

### Muricauda enzymes degrade β-mannan and β-glucan from microalgae

**Fig. 5:**
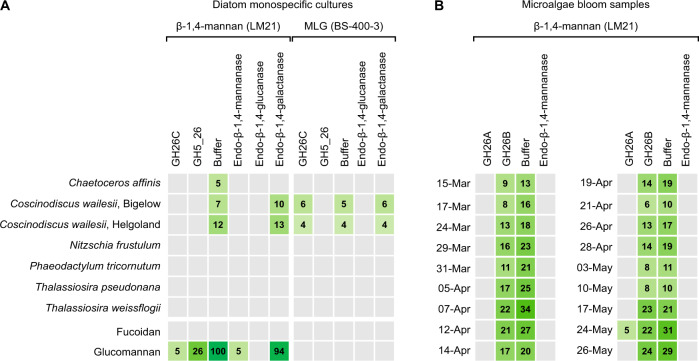
β-1,4-mannan and β-1,3/1,4-glucan substrates targeted by *Muricauda* sp. enzymes can be found in different diatom isolates. Two types of microarrays were used for epitope deletion experiments. **A** Polysaccharides from different diatom species were extracted and those extracts were printed onto microarrays. **B** High molecular weight dissolved organic matter (HMWDOM) was sampled during a diatom-dominated microalgae bloom in 2016 at the North Sea, dates are specified at the left of the sub-heatmaps. Polysaccharides from the HMWDOM were extracted and printed onto microarrays. **A**, **B** Identical arrays were treated with either recombinant *Muricauda* sp. MAR_2010_75 GH26C, GH5_26, GH26A, GH26B, buffer (control) or commercial enzymes (Megazyme). After treatment, arrays were incubated with the antibody LM21 specific to β-1,4-mannan or with the antibody BS-400-3 specific to β-1,3/1,4-glucan (mixed-linkage glucan, MLG). For both array types, signals obtained in the NaOH-extracts are shown. Values in the heatmaps correspond to the mean signal intensities of a minimum of two replicates (each extract represented by at least two spots in each array). The highest mean signal in each of the datasets was set to 100 and all other values were normalized accordingly.

Since the bacterium containing the investigated β-mannan PUL was isolated in the German Bight during a phytoplankton bloom, we hypothesized its targeted substrate originates from microalgae. In order to investigate this, we performed epitope deletion assays. Single carbohydrate microarrays (each containing microalgae-related extracts) were treated with either recombinant *Muricauda* enzymes, buffer (control) or commercial enzymes. This assay is based on the fact that an antibody binds to one specific polysaccharide epitope, thus if an enzyme hydrolyzes the polysaccharide then the epitope will be lost and the antibody binding will be reduced or completely abolished. We treated the abovementioned arrays populated with diatom extracts. We observed mannan epitope deletion when the array-immobilized diatom extracts were treated with GH26C from the PUL compared with the buffer-treated control, demonstrating this enzyme targeted the microalgal β-mannan (Fig. 5A). The monoclonal antibody (mAb) LM21 is an anti-β-1,4-mannan probe and binds to β-1,4-homomannan, glucomannan and galactomannan [57]. The abolishment of LM21 signal when treating the array with GH5_26 and with a β-1,4-glucanase, suggests that *Chaetoceros affinis* and *Coscinodiscus wailesii* produce glucomannan. Note that the commercial β-glucanase has reported activity against glucomannan and β-glucan, as described by the manufacturer (Megazyme). The LM21 signal decrease in *C. affinis* extract treated with β-galactanase may be due to certain enzyme side activity, as none of the known mannan types contains β-linked galactose. A standard glucomannan from *Konjac* was included as control and the two *Muricauda* enzymes catalyzed its degradation. Moreover, GH5_26 digested β-1,3/1,4-glucan from *C. wailesii*, as shown by the loss of signal for mAb BS-400-3 [58] compared to the buffer control (Fig. 5A). The two polysaccharides were only detected in the NaOH extracts, which is the solvent commonly used to release hemicelluloses in plants [59, 60], suggesting they might be part of the diatom cell wall.

We treated arrays populated with extracts of HMWDOM sampled during a spring diatom-dominated microalgae bloom at the North Sea. The HMWDOM extracts contained β-1,4-mannan, as shown by binding of mAb LM21 (Fig. 5B, third column). Presence of β-mannan in the dissolved fraction could be a result of diatom decay. Alternatively, it may be that diatoms contain β-1,4-mannan as part of their cell wall but also actively release some of it into the surrounding seawater. Treatment of the extracts with GH26B showed minor signal decrease compared to the control, thus no clear activity was observed for this enzyme. In contrast, when treating the extracts with GH26A the LM21 signals were abolished. While GH26A showed no activity on commercial β-mannan polysaccharide or oligosaccharide substrates, it showed activity on a β-1,4-mannan present in HMWDOM from a microalgae bloom dominated by diatoms, supporting the hypothesis that the commercially available manno-oligosaccharides were not the correct substrate (Fig. 5B).

Overall, our data support diatom β-mannan and β-glucan were targeted by widely conserved enzymes belonging to the *Muricauda* β-mannan PUL. Additionally, the epitope deletion shown by our enzymes as well as by commercial GHs further underline the presence of these two polysaccharides in marine diatoms.

## Discussion

Diatom cell wall polysaccharides represent an important yet underexplored carbon pool. Besides the specific structures of these microalgal sugar compounds, the biochemical mechanisms bacteria use to break down these polymers remain poorly understood. Based on our results, the process of bacteria-driven marine β-mannan degradation is evolutionarily and functionally similar to terrestrial gut bacteria with regards to both, enzymatic reactions and protein structures. Genomic content of experimentally determined PULs and predicted biochemical activities of our PUL-encoded enzymes align with those of gut microbiome pathways [21]. On the other hand, there also are specific enzyme adaptations to more complex marine β-mannan sources.

**Fig. 6:**
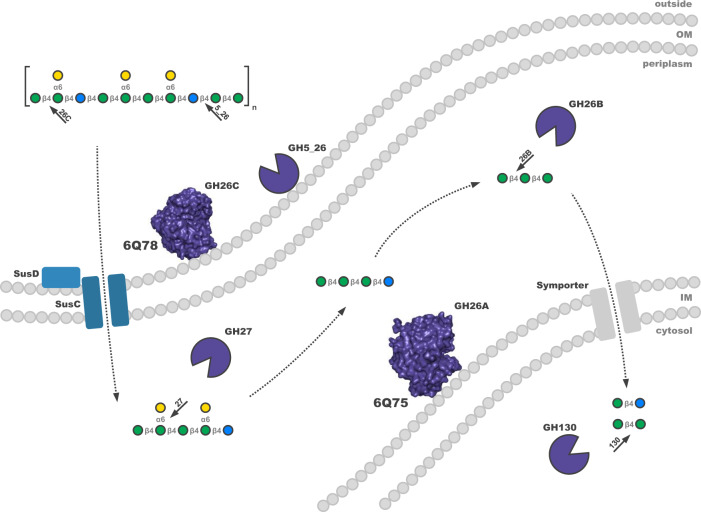
Schematic of the proposed enzymatic cascade for the degradation of galactoglucomannan substrates by *Muricauda* sp. MAR_2010_75. Key proteins and enzymes of the pathway coded for in the β-mannan PUL (FG28_RS02275-FG28_RS02380) are colored according to Fig. 2. The polysaccharide substrate sketch is shown according to Fig. 3. Regulatory elements and enzymes directed at monosaccharide processing and deacetylation were omitted for clarity. Localization of proteins was predicted via subproteome analyses by the pSORTb 3.0 and CELLO tools [42, 43]. GH5_26, GH26A, GH26C and GH27 were assigned functions based on biochemical and/or structural evidence. Functions of other proteins and CAZymes were assigned via a combined BLAST and HMMER search [66, 67]. IM inner membrane, OM outer membrane.

Based on the proteomic and biochemical data of this study, we propose an enzymatic cascade for the degradation of β-mannan-containing substrates by *Muricauda* sp. MAR_2010_75 (Fig. 6). Our subproteome analysis revealed the PUL-encoded enzymes GH26C and GH5_26 to be most abundant in the extracellular protein fraction, indicating a position attached to but not embedded in the outer membrane. GH26C, the main conserved mannanase of the PUL, was experimentally shown to be an endo-acting β-1,4 mannanase, producing oligosaccharides of varying lengths when incubated with galactomannan. The solved structure shows the classic hydrolase TIM-barrel conformation and shares active residues with structural and functional homologs of terrestrial ß-mannan-degrading bacteria [21, 48]. Homologs of this enzyme are found in similarly structured PULs of bacteroidetes found in marine databases as well as bloom isolates, underlining the importance of β-mannan-containing substrates (Fig. 1, Fig. S1). GH5_26 showed β-1,4 glucanase activity on substrates containing different β-linkages as well as activity on glucomannan, indicating that the polysaccharide targeted by this enzyme contains at least some amounts of glucose as described for terrestrial glucomannans [17]. A limitation of our study is the use of terrestrial mannans as substrates because beta-mannans from marine micro- or macroalgae are not yet available. It should be considered that the terrestrial mannans and the oligos used here for bacterial growth and enzymology are not the optimal substrates. Slow growth and incomplete digestion support this argument suggesting that the PUL with its enzymes could be targeting algal mannans with similar but also different structures compared to terrestrial mannans.

In the periplasm, further depolymerization is facilitated by an exo-α-1,6 galactosidase of family GH27 able to release galactose from β-1,4-mannan oligosaccharide backbones of different length. GH26A, which was also predicted to be present in the periplasm, showed no activity on tested manno-oligosaccharides but revealed activity on β-1,4-mannan produced during a microalgae bloom. Structural analysis revealed an additional loop blocking the +2 site of the active site that cannot be observed in homologs with high structural similarities [49, 61]. This suggests a narrower substrate specificity for which we could not determine a suitable oligosaccharide. From the other deduced enzyme activities encoded by the PUL it can, however, be speculated that the substrate is an oligosaccharide containing both mannose and glucose. Activities like this have previously been demonstrated for family 26 glycoside hydrolases [62] but additional data is needed to test this hypothesis.

Whereas proteome analyses indicated the SusC (FG28_RS02370) to channel oligosaccharides into the cell, a PUL-enocoded symporter (FG28_RS02300) transports manno- and manno-gluco-disaccharides into the cytosol. Here, they are targeted by a predicted GH130 phosphorylase, allowing the released monosaccharides to enter glycolysis [63].

The presence of an expressed transcription factor of the AraC-type (FG28_RS02325) together with the regulatory up- and downstream boundaries of the PUL show this cluster to be a genomic island for the degradation of β-mannan. The upregulation of the PUL, observed for three tested mannans (galactomannan, glucomannan and homomannan), was highest on galactomannan and glucomannan (Table S3). The abundance of a PUL-encoded predicted esterase in the membrane fraction suggests the substrate to be modified by acetylation, which is common for β-mannans [19, 20].

β-mannans, especially galactoglucomannans, have been widely characterized as a main group of hemicelluloses in plants [19, 64]. Terrestrial mannans such as galactomannan are capable of activating not only the β-mannan PUL of *Muricauda* sp. MAR_2010_75 but, as we previously showed, also similar PULs of marine bloom isolates such as those of *Salegentibacter* sp. Hel_1_6 and *Leeuwenhoekiella* sp. MAR_2009_132 [22]. With the comparative genomics of this study, we show that our proposed degradation pathway is conserved in many marine bacteria as well as bloom-associated isolates. Observed CAZyme modularities that differ from the *Muricauda* sp. PUL also suggest a β-mannan-containing substrates to show a degree of variability (Fig. 1, Fig S1).

Only recently, we detected a β-mannan of microalgal origin in HMWDOM and POM [15]. In the present study, we now for the first time link these β-mannan-targeting PULs of marine bacteria to a specific microalgae source. By analyzing carbohydrates extracted from different diatom species relevant to microalgae blooms, we showed the existence of β-1,4-mannan in *C. affinis* and in two different isolates of C. *wailesii*. This diatom-based β-mannan was degraded by recombinant versions of the GH26C and GH5_26 of *Muricauda* sp. MAR_2010_75. Furthermore, the HMWDOM β-mannan from a natural diatom bloom was degraded by a recombinant version of the GH26A. These enzymatic activities demonstrate that the enzymes conserved in PULs of different marine isolates target microalgal β-mannan.

In conclusion, we show that the β-mannan PUL of *Muricauda* sp. MAR_2010_75 can be viewed as a model for how marine bacteria target β-mannan-containing substrates. Parts of this general degradation mechanism are similar to those of gut bacteria but also bear significant differences, such as the addition of the GH5_26 (Fig. 6). The detected β-mannan links *C. affinis* and *C. wailesii* to marine bacteria containing β-mannan PULs such as *Muricauda* sp. MAR_2010_75. The specificity of this glycan-enzyme link at species resolution uncovers hypothetical connections between primary producers and secondary consumers of organic matter in the carbon cycle.

## Supplementary information

Supporting Information

Table S3

Table S4

## Data Availability

Proteome data generated and analyzed during the current study are available through the ProteomeXchange Consortium (http://proteomecentral.proteomexchange.org) via the PRIDE partner repository [44] with the identifier PXD033586. All other data generated or analyzed during this study are included in this published article and its supplementary information files.
