# Supplementary material for: Marine bacteroidetes use a conserved enzymatic cascade to digest diatom β-mannan
Source: ISME J. 2022 Nov 21;17(2):276–85. doi: 10.1038/s41396-022-01342-4 (PMC9860051; doi:10.1038/s41396-022-01342-4)
Supplement: Supplementary file 1 — Supporting Information [file 41396_2022_1342_MOESM1_ESM.pdf]

Supporting Information

Marine bacteroidetes use a conserved enzymatic cascade to digest diatom β-mannan

Irena Beidler[1±], Craig S Robb[2,3±], Silvia Vidal-Melgosa[2,3], Marie-Katherin Zühlke[1,4], Daniel Bartosik[1,4], Vipul Solanki[2,3], Stephanie Markert[1,4], Dörte Becher[5], Thomas Schweder[1,4]*, Jan-Hendrik Hehemann[2,3]*

[1] Pharmaceutical Biotechnology, Institute of Pharmacy, University Greifswald, 17489 Greifswald, Germany

[2] Max Planck Institute for Marine Microbiology, 28359 Bremen, Germany

[3] University of Bremen, Center for Marine Environmental Sciences, MARUM, 28359 Bremen, Germany

[4] Institute of Marine Biotechnology, 17489 Greifswald, Germany

[5] Institute of Microbiology, University Greifswald, 17489 Greifswald, Germany.

[±] Authors contributed equally to this work

*Corresponding authors

Thomas Schweder, e-mail: schweder@uni-greifswald.de

Jan-Hendrik Hehemann, e-mail: jheheman@mpi-bremen.de

This Supporting Information file contains:

Supplementary Methods with references

Supplementary Tables S1 - S2, S5

Supplementary Figure S1 – S5

Supplementary Tables S3 & S4 are available as separate Excel files

Supplementary Methods

Protein extraction and subproteome enrichment

Triplicates of 100 mL cultures were harvested via centrifugation (4 000 x *g*, 20 min). Cells were washed twice with TE buffer (10 mM Tris-HCl (pH 7.5), 10 mM EDTA (pH 8.0)) and resuspended in 50 mM Tris-HCl (pH 7.5) containing Roche ‘cOmplete Protease Inhibitor (Lysis Buffer) (Roche, Basel, Switzerland). Cells were disrupted via sonication (3 x 30 s) (HD/UV 2070, Bandelin, Berlin, Germany) and cell debris was removed by centrifugation. Integral membrane and cytosolic proteins for subproteomes from glucomannan cultures were separated by ultracentrifugation at 100 000 x *g* and 4 °C for 65 min. The pelleted membrane was resuspended in TE buffer and thoroughly ground using a glass homogenizer.

For the extraction of the extracellular fraction, the culture supernatant was filtered using a 0.2 µm filter to remove remaining cells and then incubated with StrataClean beads at 4 °C and 150 rpm over night to bind cell-detached protein [1]. The mixture was centrifuged at 10 000 x *g* and 4 °C and washed once with TE buffer. Remaining buffer was evaporated using a SpeedVac (Concentrator Plus, Eppendorf, Hamburg, Germany).

Protein concentration of all samples except the extracellular fraction was determined using the Pierce™ BCA Protein Assay Kit (ThermoFischer Scientific, Waltham, MA, USA). 25 µg of protein were loaded on a 10% 1D-SDS polyacrylamide gel and separated for 90 min at 120 V. After fixing with 40% ethanol/10% acetic acid followed by Coomassie G-250 staining [2], the proteins were in-gel digested overnight (16 h) using trypsin [3].

Microarray analysis

The printed arrays were first blocked for 1 h with phosphate-buffered saline (PBS) containing 5% (w/v) low fat milk powder (MPBS). The MPBS was washed three times with PBS. Then single arrays were individually incubated with either: recombinant GH26C or GH5_26 at 5 µg/mL, or 100 mM sodium phosphate buffer pH 7.0, or β-mannanase (E-BMACJ), cellulase

(E-CELTR) or β-galactanase (E-GALCJ) from Megazyme at 1 U/mL, at 37 °C and 100 rpm overnight. After the treatment, arrays were extensively washed with PBS followed by 2 h incubation with the primary monoclonal antibodies (mAbs) LM21 (PlantProbes, UK) and BS-400-3 (BioSupplies, Australia) diluted 1/10 and 1/1000 in MPBS, correspondingly. After a washing step with PBS, arrays were incubated for 2 h with the anti-rat or anti-mouse secondary antibodies conjugated to alkaline phosphatase (A8438 and A3562, Sigma-Aldrich) both diluted 1/5000 in MPBS. After washing with PBS and MilliQ, arrays were developed with alkaline phosphatase substrate. The developed arrays were analyzed as described previously [4]. The highest mean signal intensity value obtained in the whole data set was set to 100 and all other values were normalized accordingly. A cut-off of 4 arbitrary units was applied. Controls for the extraction solvents and for the secondary antibodies showed no unspecific binding of the probes.

Table S1. Information on diatom species used for the search of marine β-mannan sources

**Diatom species**	**Strain information**
Chaetoceros affinis	CCMP158 (NCMA, Bigelow Laboratory)
Coscinodiscus wailesii (strain 1)	CCMP2513 (NCMA, Bigelow Laboratory)
Coscinodiscus wailesii (strain 2)	Isolated at Helgoland (54˚11.3′N,7˚54.0′E), North Sea
Nitzschia frustulum	CCMP558 (NCMA, Bigelow Laboratory)
Phaeodactylum tricornutum	CCMP2561 (NCMA, Bigelow Laboratory)
Thalassiosira pseudonana	provided by André Scheffel (Potsdam, Germany)
Thalassiosira weissflogii	provided by André Scheffel (Potsdam, Germany)

Table S2. Primer sequences

Name	Protein ID	Sequence
GH5_fw	WP_197062539.1	GTTTAACTTTAAGAAGGAGATATACCATGGATGGGGCGTATATCAGTCGATG
GH5_rv	WP_197062539.1	ATCTCAGTGGTGGTGGTGGTGGTGCTCGAGCTTATTGCCCTTGTCATAGCTCTG
GH27_fw	WP_036379578.1	ATCTCAGTGGTGGTGGTGGTGGTGCTCGAGAAATCTATTCTTTTTCTCAAGCAACACCATT
GH27_rv	WP_036379578.1	GTTTAACTTTAAGAAGGAGATATACCATGGTGTGGTCAAAAATTTAATGGGCTTGCC
GH26A_fw	WP_036379595.1	GTTTAACTTTAAGAAGGAGATATACCATGGTCAATGGGAATGCCACGGAAG
GH26B-fw	WP_197062540.1	GTTTAACTTTAAGAAGGAGATATACCATGGGCAATACCTCCCTCACCGACA
GH26B_rev	WP_197062540.1	ATCTCAGTGGTGGTGGTGGTGGTGCTCGAGTTTAATTTTTTTGATATCCTTTAAAAACA
GH26A_rv	WP_036379595.1	ATCTCAGTGGTGGTGGTGGTGGTGCTCGAGGCGCTCGTTGGTAAACTGTATCTCATC
GH26C_fw	WP_036379585.1	GTTTAACTTTAAGAAGGAGATATACCATGGGAAGTTTCATGGTAAACCCGGATG
GH26C_rv	WP_036379585.1	ATCTCAGTGGTGGTGGTGGTGGTGAGAATTGGGCGGTAGCCGGTAC

Table S3. Whole cell proteomics results. *Muricauda* sp. was grown on homomannan, galactomannan, glucomannan, mannose and citrus pectin as sole carbon source. Only proteins that could be detected in at least two of three replicates were considered identified. Automatically calculated iBAQ values were used to manually calculate % riBAQ for semiquantitative comparisons between samples from different conditions. Tests for differential expression were performed using Perseus v. 1.6.2.3 [5] with Welch's two-sided t-test (permutation-based FDR 0.05). (see separate Excel file)

Table S4. Subproteomics results with location prediction. Tests for differential expression were performed using Perseus v. 1.6.2.3 [5] with Welch's two-sided t-test (permutation-based FDR 0.05). Localization of the proteins was additionally analyzed via the pSORTb 3.0 and CELLO tools [6, 7]. (see separate Excel file)

Table S5. X-ray data collection, processing and model refinement statistics for M_GH26C and M_GH26A

**Dataset**	**GH26A**	**GH26C**
Resolution (Å)	45.12-1.75 (1.84-1.75)	49.40-1.50 (1.52-1.50)
Space group	P1	C121
Unit Cell (Å) (˚)	47.524, 49.342, 71.711 (77.999, 89.831, 69.641)	94.119, 60.358, 148.99 (90, 95.925, 90)
No. of Reflections	248361 (36556)	884001 (39440)
No. Unique	58106 (8362)	132761 (6137)
R_{sym} (%)	0.089 (0.482)	0.117 (0.945)
Completeness (%)	9.2 (2.7)	9.5 (2.2)
Redundancy	96.7 (95.2)	99.5 (93.6)
<*I*/□(*I*)>	4.3 (4.4)	6.7 (6.4)
Mosaicity	0.13	0.06
Refinement		
R_{work}/R_{free} (%)	15/19	16/19
Number of Atoms	5418	6747
Protein	4970	6189
Ligand (TRS; GOL)	20	18.3
Water	428	842
B factors		
Overall	26.09	18.3
Protein	25.12	16.49
Ligand (TRS; GOL)	32.04	18.68
Water	37.01	31.23
RMSZ		
Bond Lengths	0.009	0.011
Bond Angles	0.95	1.1
Ramachandran Statistics (%)		
Favored	96.4	97.33
Allowed	3.56	2.4
Outliers	0	0.27
PDB accession code	6Q75	6Q78

Supplementary Figures

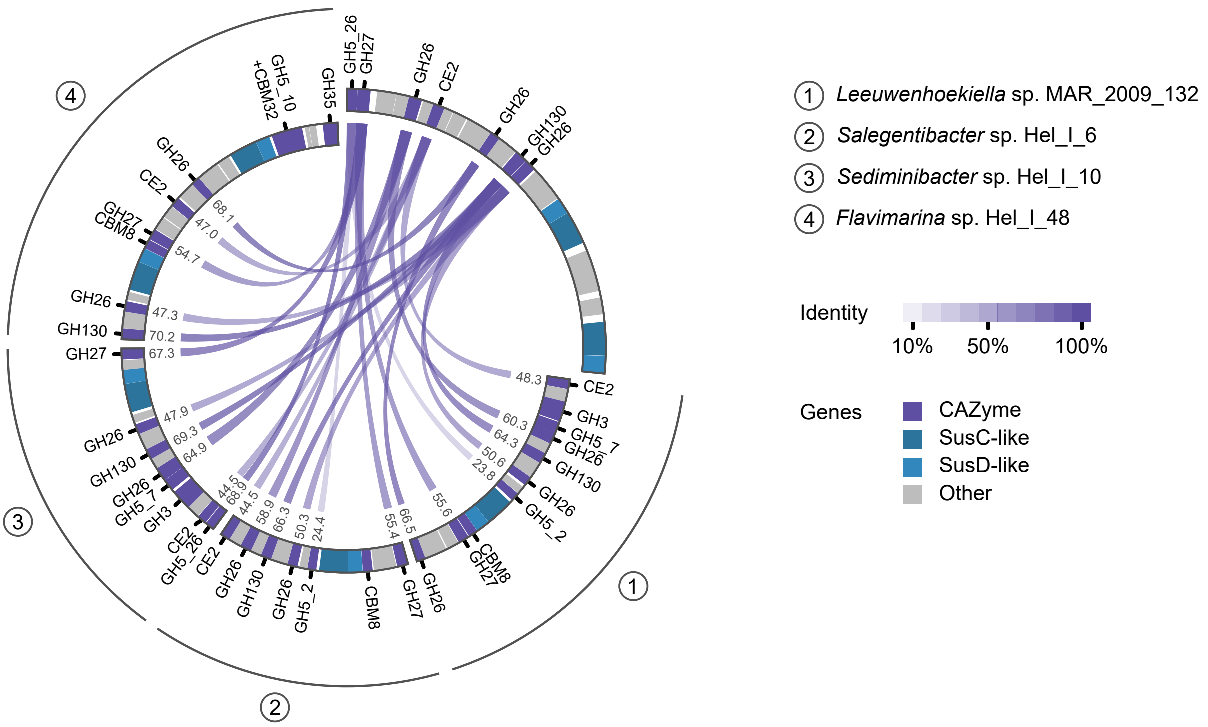

Figure S1. CAZyme synteny of the *Muricauda* sp. MAR_2010_75 β-mannan PUL with those of other bloom-associated strains. Only the highest scoring hit for each CAZyme in a PUL is shown. Ribbon color intensity indicates degree of similarity; cutoff 10^{-5}. Identities (%) are given for each link.

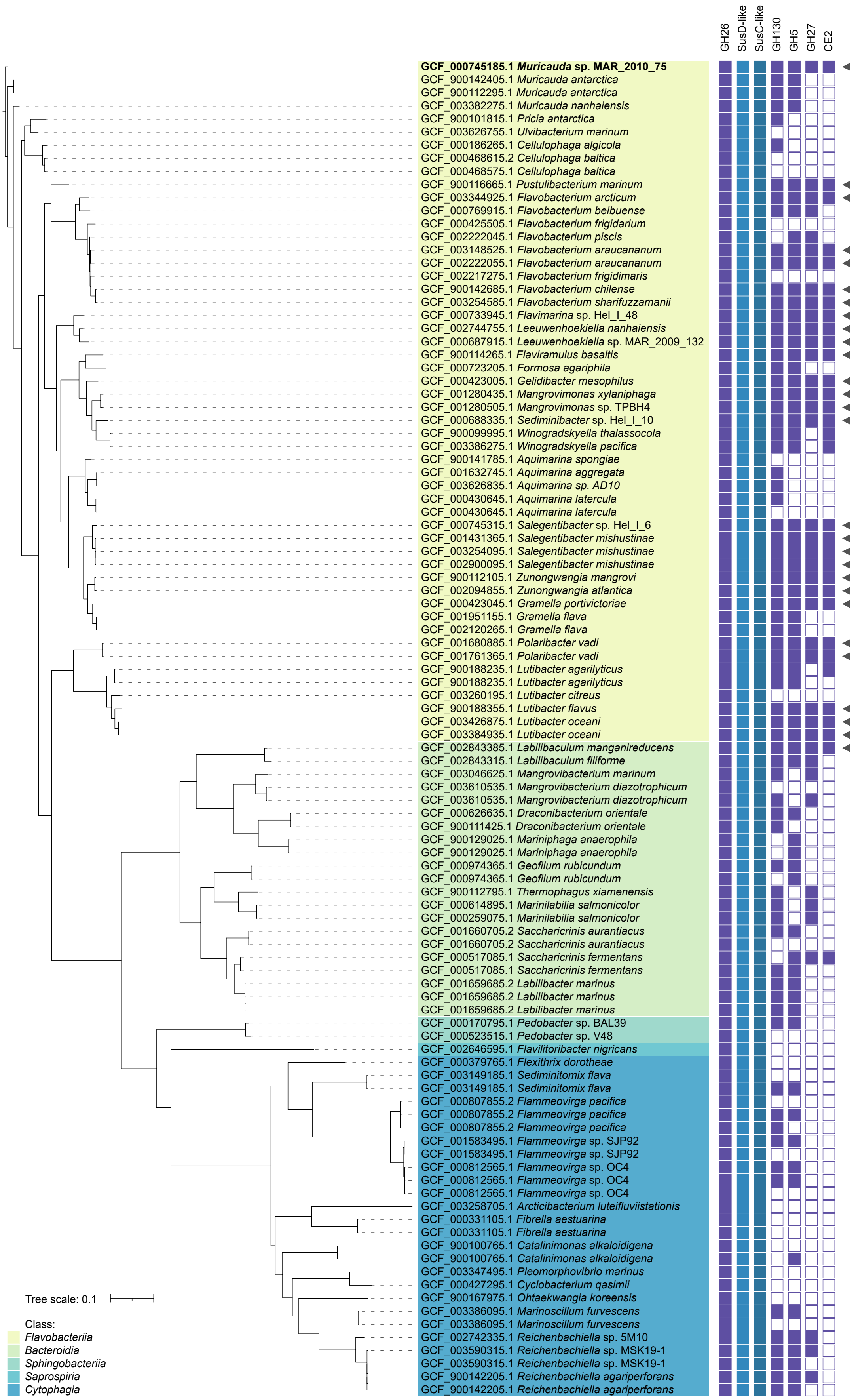
GH26
SusD-like
SusC-like
GH130
GH5
GH27
CE2
GCF_000745185.1 Muricauda sp. MAR_2010_75
GCF_900142405.1 Muricauda antarctica
GCF_900112295.1 Muricauda antarctica
GCF_003382275.1 Muricauda nanhaiensis
GCF_900101815.1 Pricia antarctica
GCF_003626755.1 Ulvibacterium marinum
GCF_000186265.1 Cellulophaga algicola
GCF_000468615.2 Cellulophaga baltica
GCF_000468575.1 Cellulophaga baltica
GCF_900116665.1 Pustulibacterium marinum
GCF_003344925.1 Flavobacterium arcticum
GCF_000769915.1 Flavobacterium beibuense
GCF_000425505.1 Flavobacterium frigidarium
GCF_002222045.1 Flavobacterium piscis
GCF_003148525.1 Flavobacterium araucananum
GCF_002222055.1 Flavobacterium araucananum
GCF_002217275.1 Flavobacterium frigidimaris
GCF_900142685.1 Flavobacterium chilense
GCF_003254585.1 Flavobacterium sharifuzzamanii
GCF_000733945.1 Flavimarina sp. Hel_I_48
GCF_002744755.1 Leeuwenhoekiella nanhaiensis
GCF_000687915.1 Leeuwenhoekiella sp. MAR_2009_132
GCF_900114265.1 Flaviramulus basaltis
GCF_000723205.1 Formosa agariphila
GCF_000423005.1 Gelidibacter mesophilus
GCF_001280435.1 Mangrovimonas xylaniphaga
GCF_001280505.1 Mangrovimonas sp. TPBH4
GCF_000688335.1 Sediminibacter sp. Hel_I_10
GCF_900099995.1 Winogradskyella thalassocola
GCF_003386275.1 Winogradskyella pacifica
GCF_900141785.1 Aquimarina spongiae
GCF_001632745.1 Aquimarina aggregata
GCF_003626835.1 Aquimarina sp. AD10
GCF_000430645.1 Aquimarina latercula
GCF_000430645.1 Aquimarina latercula
GCF_000745315.1 Salegentibacter sp. Hel_I_6
GCF_001431365.1 Salegentibacter mishustinae
GCF_003254095.1 Salegentibacter mishustinae
GCF_002900095.1 Salegentibacter mishustinae
GCF_900112105.1 Zunongwangia mangrovi
GCF_002094855.1 Zunongwangia atlantica
GCF_000423045.1 Gramella portivictoriae
GCF_001951155.1 Gramella flava
GCF_002120265.1 Gramella flava
GCF_001680885.1 Polaribacter vadi
GCF_001761365.1 Polaribacter vadi
GCF_900188235.1 Lutibacter agarilyticus
GCF_900188235.1 Lutibacter agarilyticus
GCF_003260195.1 Lutibacter citreus
GCF_900188355.1 Lutibacter flavus
GCF_003426875.1 Lutibacter oceani
GCF_003384935.1 Lutibacter oceani
GCF_002843385.1 Labilibaculum manganireducens
GCF_002843315.1 Labilibaculum filiforme
GCF_003046625.1 Mangrovibacterium marinum
GCF_003610535.1 Mangrovibacterium diazotrophicum
GCF_003610535.1 Mangrovibacterium diazotrophicum
GCF_000626635.1 Draconibacterium orientale
GCF_900111425.1 Draconibacterium orientale
GCF_900129025.1 Mariniphaga anaerophila
GCF_900129025.1 Mariniphaga anaerophila
GCF_000974365.1 Geofilum rubicundum
GCF_000974365.1 Geofilum rubicundum
GCF_900112795.1 Thermophagus xiamenensis
GCF_000614895.1 Marinilabilia salmonicolor
GCF_000259075.1 Marinilabilia salmonicolor
GCF_001660705.2 Saccharicrinis aurantiacus
GCF_001660705.2 Saccharicrinis aurantiacus
GCF_000517085.1 Saccharicrinis fermentans
GCF_000517085.1 Saccharicrinis fermentans
GCF_001659685.2 Labilibacter marinus
GCF_001659685.2 Labilibacter marinus
GCF_001659685.2 Labilibacter marinus
GCF_000170795.1 Pedobacter sp. BAL39
GCF_000523515.1 Pedobacter sp. V48
GCF_002646595.1 Flavilitoribacter nigricans
GCF_000379765.1 Flexithrix dorotheae
GCF_003149185.1 Sediminitomix flava
GCF_003149185.1 Sediminitomix flava
GCF_000807855.2 Flammeovirga pacifica
GCF_000807855.2 Flammeovirga pacifica
GCF_000807855.2 Flammeovirga pacifica
GCF_001583495.1 Flammeovirga sp. SJP92
GCF_001583495.1 Flammeovirga sp. SJP92
GCF_000812565.1 Flammeovirga sp. OC4
GCF_000812565.1 Flammeovirga sp. OC4
GCF_000812565.1 Flammeovirga sp. OC4
GCF_003258705.1 Arcticibacterium luteifluviistationis
GCF_000331105.1 Fibrella aestuarina
GCF_000331105.1 Fibrella aestuarina
GCF_900100765.1 Catalinimonas alkaloidigena
GCF_900100765.1 Catalinimonas alkaloidigena
GCF_003347495.1 Pleomorphovibrio marinus
GCF_000427295.1 Cyclobacterium qasimii
GCF_900167975.1 Ohtaekwangia koreensis
GCF_003386095.1 Marinoscillum furvescens
GCF_003386095.1 Marinoscillum furvescens
GCF_002742335.1 Reichenbachiella sp. 5M10
GCF_003590315.1 Reichenbachiella sp. MSK19-1
GCF_003590315.1 Reichenbachiella sp. MSK19-1
GCF_900142205.1 Reichenbachiella agariperforans
GCF_900142205.1 Reichenbachiella agariperforans
Tree scale: 0.1
Class:
Flavobacteriia
Bacteroidia
Sphingobacteriia
Saprospiria
Cytophagia

Figure S2. Maximum likelihood phylogeny of all clusters similar to the *Muricauda* sp. MAR_2010_75 PUL found in marine databases. Class of the bacteria encoding for the found clusters is given in color. The modularity of each cluster as it compares to the *Muricauda* sp. PUL is shown on the right.

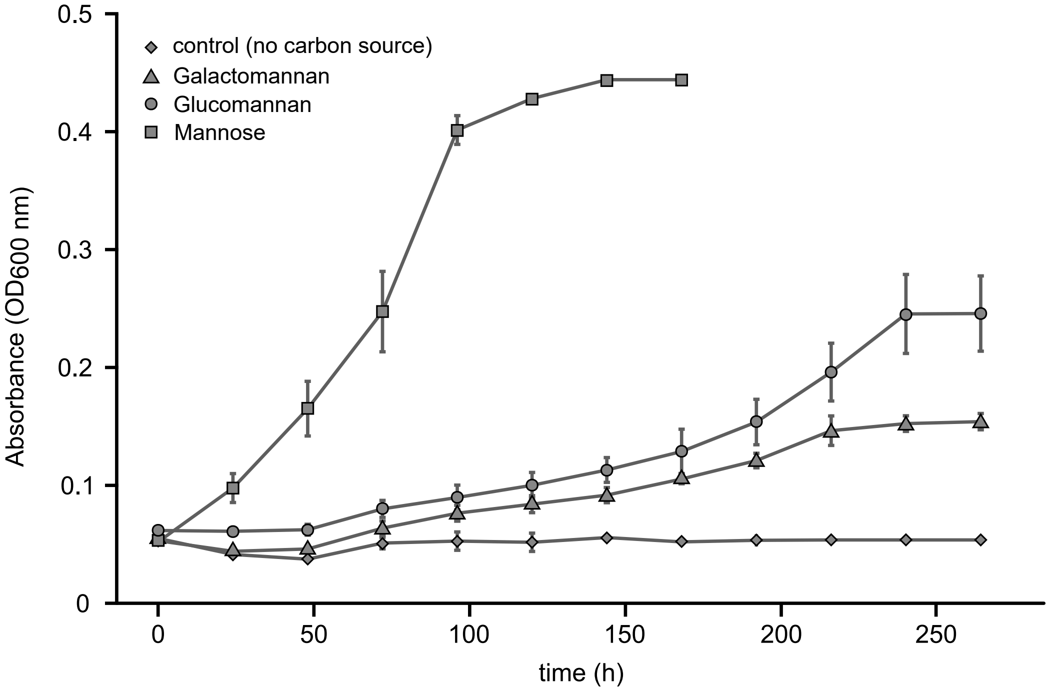

Figure S3. *Muricauda* sp. MAR_2010_75 is able to use multiple mannose-containing substrates as sole carbon source. The strain was grown in MPM-medium with 0.2% (w/v) specific poly- or monosaccharides until no significant changes in optical density could be detected over the course of 24 h after exponential growth (time points omitted). Values shown depict the mean of three replicates with standard deviation.

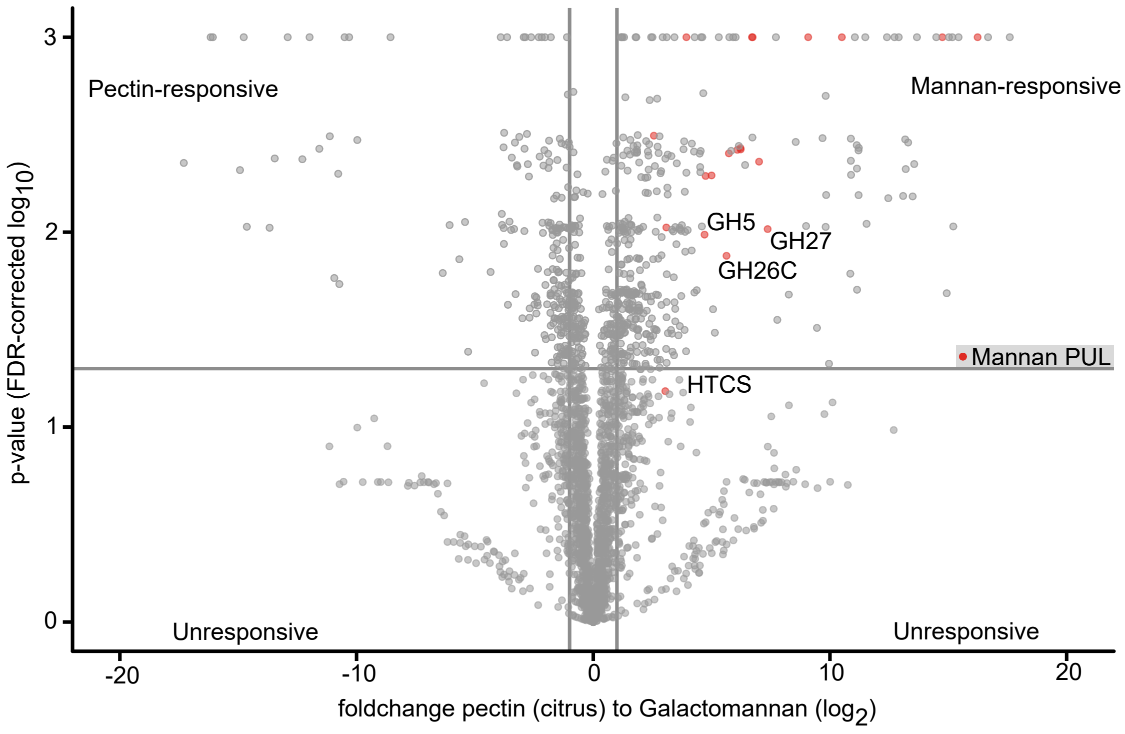

Figure S4. Growth on β-mannan-containing substrates specifically induces proteins of the *Muricauda* sp. MAR_2010_75 mannan-PUL in a statistically significant manner. Fold changes and p-values were calculated from MS-measurements of three biological replicates per substrate using the Perseus statistical software [5]. Proteins not detected in one condition were given an artificial value 1.25 times lower than the lowest detected value in the sample to show their significance. Proteins belonging to the β-mannan PUL are shown in red.

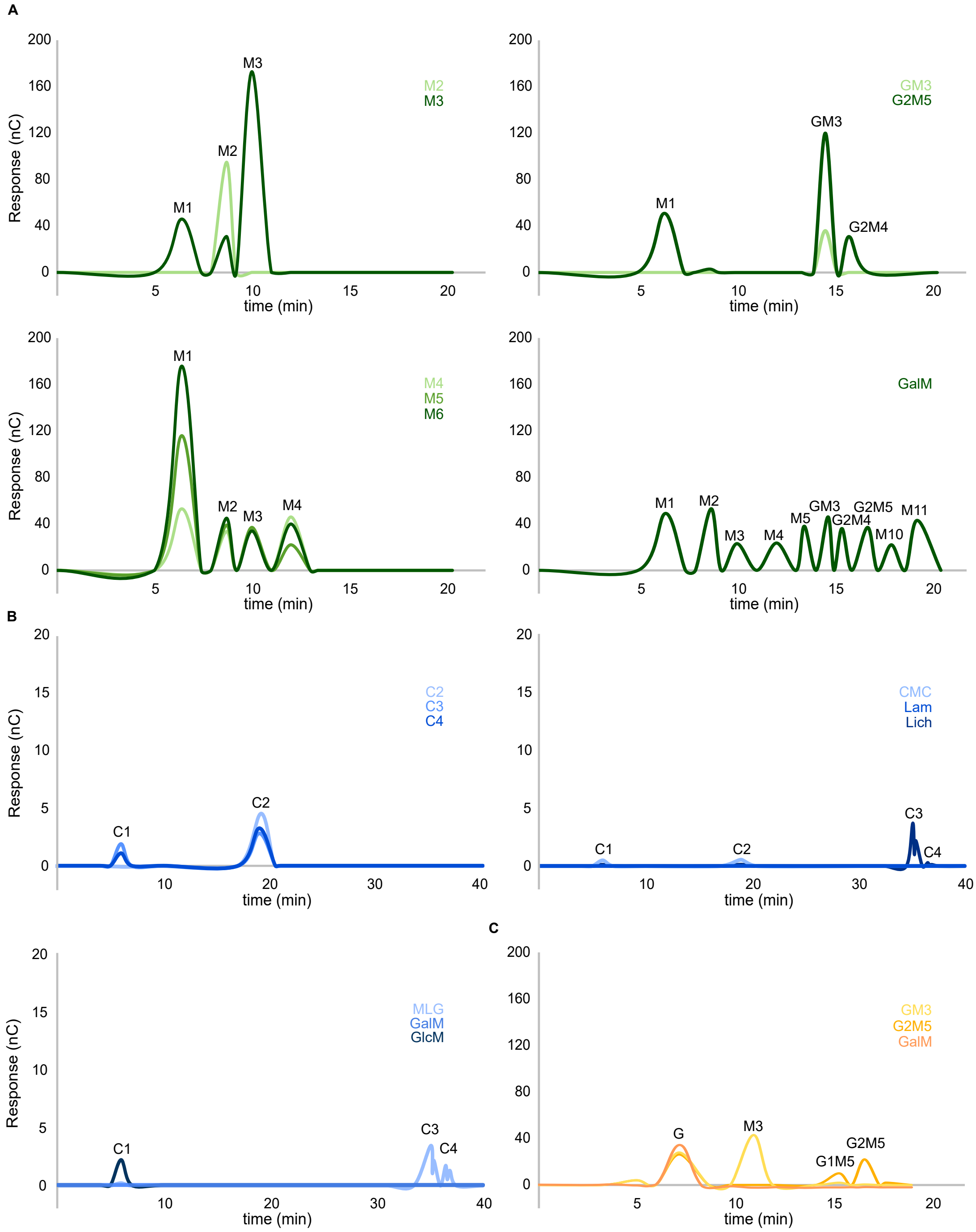

Figure S5. HPAEC-PAD data of recombinantly produced enzymes with different poly- and oligosaccharides. Enzymes GH26C (A), GH5_26 (B) and GH27 (C) were incubated with the respective substrate (in color) for 2 h. Samples were heat inactivated and then measured diluted 1:1000. Observed peaks were compared to mono- and oligosaccharide standards (black). M manno-, C cellu-, G galactomono or -oligosaccharides with dp given as the number behind them.

References

1. Bonn F, Otto A (2018) Protein Enrichment from Highly Dilute Samples with StrataClean. Methods Mol Biol 1841:11–18. https://doi.org/10.1007/978-1-4939-8695-8_2
2. Candiano G, Bruschi M, Musante L et al. (2004) Blue silver: a very sensitive colloidal Coomassie G-250 staining for proteome analysis. Electrophoresis 25:1327–1333. https://doi.org/10.1002/elps.200305844
3. Grube M, Cernava T, Soh J et al. (2015) Exploring functional contexts of symbiotic sustain within lichen-associated bacteria by comparative omics. ISME J 9:412–424. https://doi.org/10.1038/ismej.2014.138
4. Vidal-Melgosa S, Sichert A, Francis TB et al. (2021) Diatom fucan polysaccharide precipitates carbon during algal blooms. Nat Commun 12:1150. https://doi.org/10.1038/s41467-021-21009-6
5. Tyanova S, Temu T, Sinitcyn P et al. (2016) The Perseus computational platform for comprehensive analysis of (prote)omics data. Nature Methods 13:731–740. https://doi.org/10.1038/nmeth.3901
6. Yu C-S, Lin C-J, Hwang J-K (2004) Predicting subcellular localization of proteins for Gram-negative bacteria by support vector machines based on n-peptide compositions. Protein Sci 13:1402–1406. https://doi.org/10.1110/ps.03479604
7. Yu NY, Wagner JR, Laird MR et al. (2010) PSORTb 3.0: improved protein subcellular localization prediction with refined localization subcategories and predictive capabilities for all prokaryotes. Bioinformatics 26:1608–1615. https://doi.org/10.1093/bioinformatics/btq249
